# Supplementary material for: Reversed HILIC Gradient: A Powerful Strategy for On-Line Comprehensive 2D-LC
Source: Molecules. 2023 May 5;28(9):3907. doi: 10.3390/molecules28093907 (PMC10179806; doi:10.3390/molecules28093907)
Supplement: Supplementary file 1 [file molecules-28-03907-s001.zip › molecules-2311065-supplementary.pdf]

Supplementary information for:

Reversed HILIC gradient: a powerful strategy for on-line comprehensive 2D-LC

[1] Université de Lyon, Institut Des Sciences Analytiques, UMR 5280, CNRS, 5 rue de la Doua, 69100 Villeurbanne, France
[2] University of Leuven (KU Leuven), Department of Pharmaceutical and Pharmacological Sciences, Pharmaceutical Analysis, Herestraat 49, Leuven, Belgium
[3] School of Pharmaceutical Sciences, University of Geneva, CMU - Rue Michel Servet 1, 1211 Geneva 4, Switzerland
[4] Institute of Pharmaceutical Sciences of Western Switzerland, University of Geneva, CMU - Rue Michel Servet 1, 1211 Geneva 4, Switzerland
* Correspondence: sabine.heinisch@univ-lyon1.fr

Figure S1: S vs. log k0 values plots for various conditions. (A) pharmaceutical compounds in RPLC, (B) pharmaceutical compounds in HILIC, (C) pharmaceutical compounds in revHILIC, (D) peptides in RPLC, (E) peptides in HILIC, (F) peptides in revHILIC.

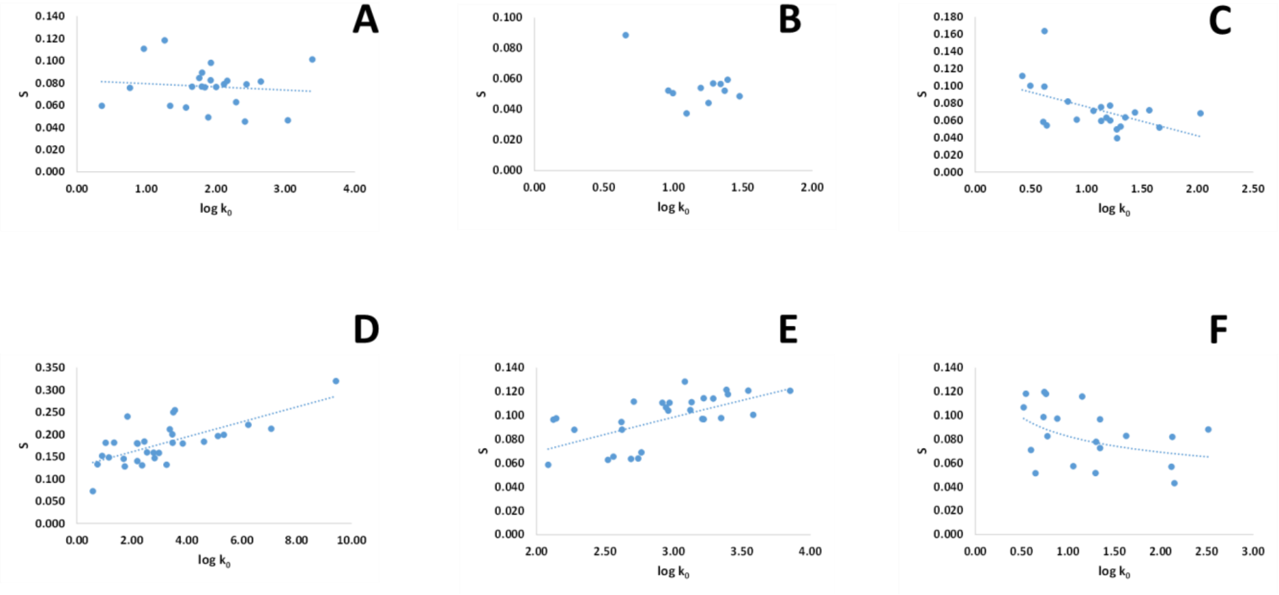

Figure S2: Overlay of numerous representative chromatograms of small drugs in three different chromatographic modes. (A) RPLC analysis with a 1 – 99 %B gradient in 2.4 min (b_{av} = 0.18). (B) HILIC analysis with a 2 – 42 %B gradient in 2.4 min (b_{av} = 0.22). (A) revHILIC analysis with a 1 – 51 %B gradient in 4.8 min (b_{av} = 0.18).

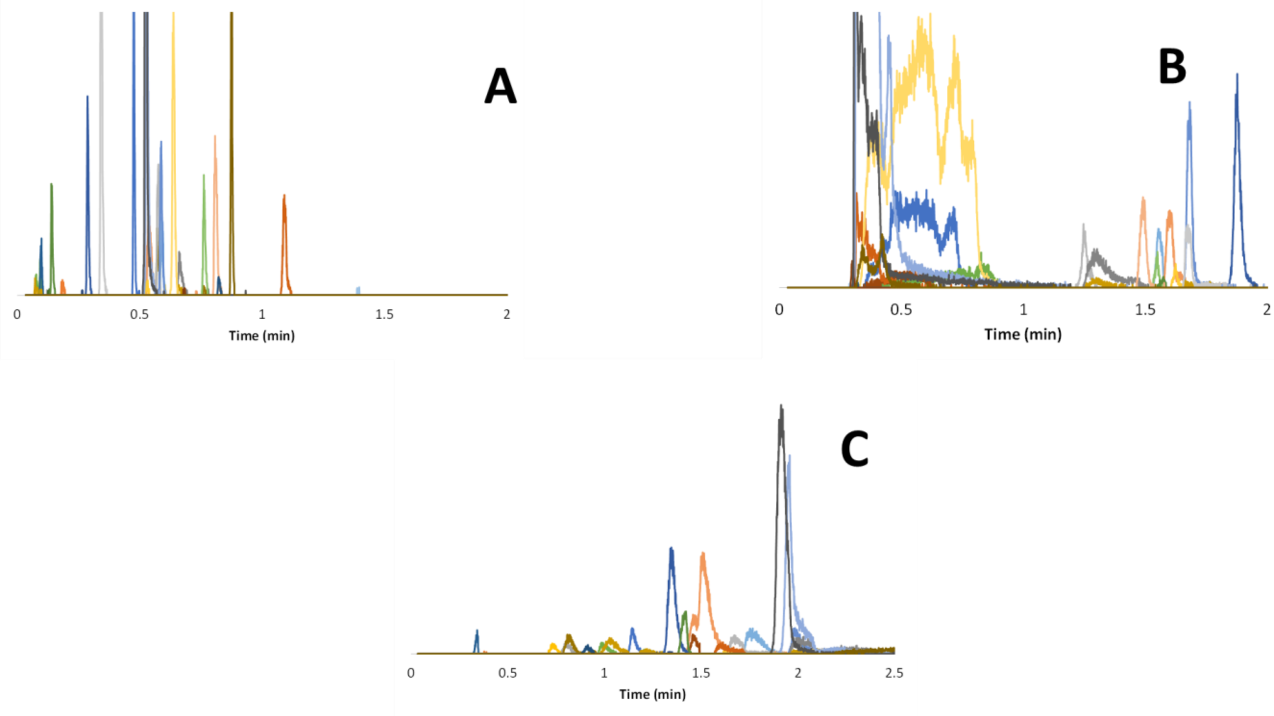

Figure S3: Determination of the retention space coverage for the three separations obtained for the representative peptide sample. (A) RPLC x RPLC, (B) HILIC x RPLC, (C) revHILIC x RPLC.

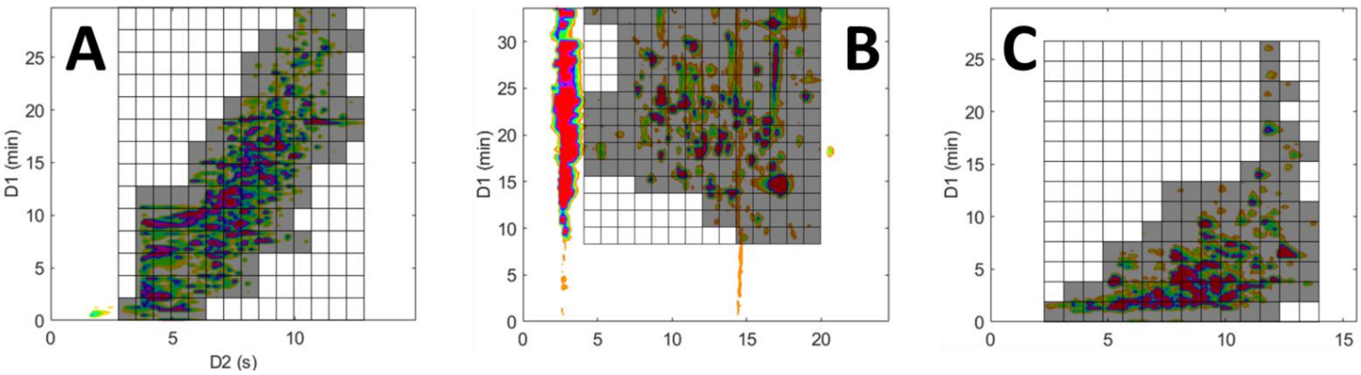

Figure S4: Determination of the retention space coverage for the three separations obtained for the representative pharmaceutical sample. (A) RPLC x RPLC, (B) HILIC x RPLC, (C) revHILIC x RPLC.

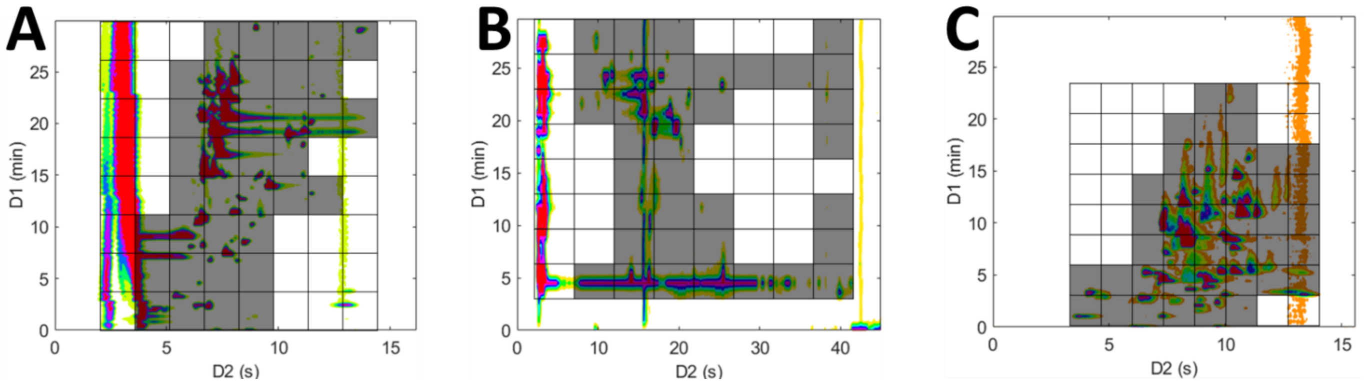

Table S1: Physical properties of the ten seven standards used in this study.

#	Peptide name	Molecular weight (g/mol)	Isoelectric point
1	Leucine enkephalin	555.62	6
2	Bombesin	1619.85	7.6
3	[arg8]-Vasopressin	1084.23	8.2
4	[ile]-Angiotensin	897.08	9.4
5	Bradykinin fragment 1-5	572.66	10.6
6	Substance P	1347.63	11.7
7	Bradykinin	1060.21	12.5

Table S2: Physico-chemical properties of the sixty-seven reference standard pharmaceuticals used in this study.

Name	Formula	pKa1	pKa2	log P	log D (pH=3)	log D (pH=9)	Mw (g/mol)
Benzoic acid	C7H6O2	4.2	-	1.895	1.87	-1.25	122.036779
Nicotinamide	C6H6N2O	14.83	3.54	-0.11	-0.75	-0.11	122.048013
Nicotinic acid	C6H5NO2	2.17	4.82	0.147	-1.23	-2.84	123.032028
Salicylic acid	C7H6O3	3.01	-	2.061	1.79	-1.09	138.031684
Glutamic acid	C5H9NO4	2.17	9.76	-1.44	-3.99	-5	147.053158
Paracetamol	C8H9NO2	9.86	1.72	0.339	0.32	0.28	151.063329
Cathine	C9H13NO	9.37	13.9	0.57	-	-	151.099714
Mandelic acid	C8H8O3	-4.1	3.75	0.66	-	-	152.047344
Methylparaben	C8H8O3	8.3	-	1.865	1.86	1.09	152.047344
Benzocaine	C9H11NO2	2.51	-	1.945	1.82	1.94	165.078979
Ephedrine	C10H15NO	10.3	-	1.13	-	-	165.115364
Pyridoxine	C8H11NO3	8.37	5.06	-1.1	-3.03	-1.87	169.073893
Sulfanilamide	C6H8N2O2S	10.1	1.85	-0.72	-0.75	-0.75	172.030648
Menadione	C11H8O2	-7.2	-	2.379	2.38	2.38	172.05243
Ascorbic acid	C6H8O6	4.37	-	-2.41	-2.43	-5.88	176.032088
Phenacetin	C10H13NO2	14.57	1.42	1.626	1.61	1.63	179.094629
Aspirin	C9H8O4	3.48	-	1.19	1.07	-1.96	180.042259
Theobromine	C7H8N4O2	9.9	0.59	-0.72	-0.72	-0.77	180.064726
Theophylline	C7H8N4O2	8.6	1.45	-0.18	-0.2	-0.71	180.064726
Saccharine	C7H5NO3S	1.6	-12.79	0.91	-0.41	-1.09	182.999014
Phenazone	C11H12N2O	0.7	-	0.268	0.27	0.27	188.094963
Cafeine	C8H10N4O2	0.73	-	-0.13	-0.13	-0.13	194.080376
Pilocarpine	C11H16N2O2	7.02	-	-0.1	-2.58	-0.1	208.121178
Sulfaguanidine	C7H10N4O2S	11.22	1.88	-1.22	-1.26	-1.23	214.052447
Meprobamate	C9H18N2O4	13.09	-1.09	0.699	0.7	0.7	218.126657
Aminophenazone	C13H17N3O	4.5	-	0.758	-0.82	0.76	231.137162
Nalidixic acid	C12H12N2O3	3.45	6.5	1.003	-0.05	-1.88	232.084792
Phenobarbital	C12H12N2O3	7.63	-	1.668	1.67	0.61	232.084792

Name	**Formula**	**pKa1**	**pKa2**	**log P**	**log D (pH=3)**	**log D (pH=9)**	**Mw (g/mol)**
Melatonine	C12H16N2O2	-1.6	15.8	1.6	-	-	232.121178
Lidocaine	C14H22N2O	14.23	8.23	2.359	-0.76	2.23	234.173213
Carbromal	C7H13BrN2O2	10.69	-0.6	1.603	1.6	1.59	236.016041
Procaine	C13H20N2O2	9.24	-	2.364	-0.78	1.93	236.152478
Nitrofurantoïne	C8H6N4O5	7.69	-2.36	-0.4	-0.4	-1.65	238.033819
Mefenamic acid	C15H15NO2	3.73	-1.31	5.33	5.25	2.18	241.2851
Sulfadiazine	C10H10N4O2S	6.5	1.64	-0.12	-0.13	-2	250.052446
Phenytoïne	C15H12N2O2	8.33	-2.81	2.524	2.52	1.79	252.089878
Diphenhydramine	C17H21NO	8.76	-	3.662	0.56	3.46	255.162314
Propranolol	C16H21NO2	13.84	9.14	3.097	0	2.72	259.157229
Tetracaine	C15H24N2O2	8.24	-	3.649	3.58	0.54	264.183778
Antazoline	C17H19N3	10.29	-	4.392	2.35	3.16	265.157898
Dextromethorphan	C18H25NO	9.85	-	3.75	-	-	271.193614
Amitryptiline	C20H23N	9.18	-	4.92	1.82	4.52	277.18305
Flufenamic acid	C14H10F3NO2	3.67	-2.51	5.62	5.53	2.47	281.066363
Nitrazepam	C15H11N3O3	2.65	11.66	2.25	-	-	281.080041
Diazepam	C16H13ClN2O	3.4	-	2.82	-	-	284.071641
Promethazine	C17H20N2S	8.98	-	4.782	1.68	4.49	284.134719
Morphine	C17H19NO3	8.21	-	0.87	-	-	285.136493
Atropine	C17H23NO3	14.11	9.98	1.528	-1.57	0.51	289.167794
Hydrochlorothiazide	C7H8ClN3O4S2	8.95	-4.08	-0.07	-0.07	-0.57	296.964475
Codeine	C18H21NO3	13.41	8.25	1.199	-1.9	1.13	299.152144
Phenylbutazone	C19H20N2O2	4.29	-0.29	3.16	3.14	-0.84	308.152478
Metamizole	C13H17N3O4S	-1.4	-0.54	-	-	-	311.093977
Chlorpromazine	C17H19ClN2S	9.41	-	5.203	2.1	4.65	318.095747
Chloramphenicol	C11H12Cl2N2O5	11.03	-1.73	1.018	1.02	1.01	322.012327
Quinidine	C20H24N2O2	12.8	9.28	3.44	-0.6	2.98	324.183778
Quinine	C20H24N2O2	12.8	9.28	3.44	-0.6	2.98	324.183778
Midazolam	C18H13CLFN3	6.19	-	3.89	-	-	325.078203
Niclosamide	C13H8Cl2N2O4	7.45	-2.36	5.405	5.4	3.85	325.986112
Furosemide	C12H11ClN2O5S	3.04	-2.49	3.001	2.72	-0.21	330.00772
Papaverine	C20H21NO4	6.32	-	3.741	1.3	3.74	339.147058
Indomethacine	C19H16ClNO4	3.96	-	3.105	3.06	-0.62	357.076786
Bisacodyl	C22H19NO4	4.41	-	2.871	1.47	2.87	361.131408
Hydrocortisone	C21H30O5	12.47	-	1.426	1.43	1.43	362.209324
Bromhexine	C14H20Br2N2	8.34	-	5.081	1.98	4.99	373.999324
Riboflavine	C17H20N4O6	4.41	-2.24	-2.02	-2.04	-4.02	376.138284
Noscapine	C22H23NO7	6.32	-	2.826	-0.07	2.82	413.147452
Reserpine	C33H40N2O9	18.11	7.25	4.046	0.98	4.04	608.273381
